# Precursor Quantitation Methods for Next Generation Food Production

**DOI:** 10.3389/fbioe.2022.849177

**Published:** 2022-03-10

**Authors:** Xinran Wang, Xiaozhou Luo

**Affiliations:** ^1^ CAS Key Laboratory of Quantitative Engineering Biology, Shenzhen Institute of Synthetic Biology, Shenzhen Institute of Advanced Technology, Chinese Academy of Sciences, Shenzhen, China; ^2^ Shenzhen Institute of Advanced Technology, Shenzhen, China

**Keywords:** precursor, quantitation, food, synthetic biology, method

## Abstract

Food is essential for human survival. Nowadays, traditional agriculture faces challenges in balancing the need of sustainable environmental development and the rising food demand caused by an increasing population. In addition, in the emerging of consumers’ awareness of health related issues bring a growing trend towards novel nature-based food additives. Synthetic biology, using engineered microbial cell factories for production of various molecules, shows great advantages for generating food alternatives and additives, which not only relieve the pressure laid on tradition agriculture, but also create a new stage in healthy and sustainable food supplement. The biosynthesis of food components (protein, fats, carbohydrates or vitamins) in engineered microbial cells often involves cellular central metabolic pathways, where common precursors are processed into different proteins and products. Quantitation of the precursors provides information of the metabolic flux and intracellular metabolic state, giving guidance for precise pathway engineering. In this review, we summarized the quantitation methods for most cellular biosynthetic precursors, including energy molecules and co-factors involved in redox-reactions. It will also be useful for studies worked on pathway engineering of other microbial-derived metabolites. Finally, advantages and limitations of each method are discussed.

## Introduction

Food is a basic element for human beings as the main energy resource for body growth and maintenance in daily life. Nowadays, food mainly comes from traditional agriculture such as planting and raising livestock. However, severer environmental problems are caused such as high greenhouse gas emission in cattle field, high water usage problems, biodiversity loss by transforming forests into farms (Fabrica 2021). Moreover, there is growing need for healthier and novel nature-based food products, such as foods without synthetic petroleum-based food dyes, or with sugar substitutes (Fabrica 2021), which cannot be satisfied by traditional agriculture.

The rise up of synthetic biology shows promise to complement the limitation of traditional agriculture, as it engineers microbial cells to be factories for production of various products (Katz et al., 2018). Since the basic food components are proteins, carbohydrates, fatty acids and vitamins (Colen et al., 2018), microbial cells can be engineered to produce each food components. These years, many progresses have been made in this area. For example, fatty acids, as a main component for meat flavor and taste used in meat analogues, was overproduced in engineered microbial cells by methods of synthetic biology (Fernandez-Moya and Da Silva 2017; Ledesma-Amaro et al., 2018; Marella et al., 2018). Linalool and geraniol, as the primary flavor components in beer previously contributed by hops, was produced in engineered yeast, to be an alternative supplementation in beer fermentation without hops addition (Denby et al., 2018). A good review has already summarized the progress of this area, which would not be discussed here in detail (Lv et al., 2021).

**FIGURE 1:**
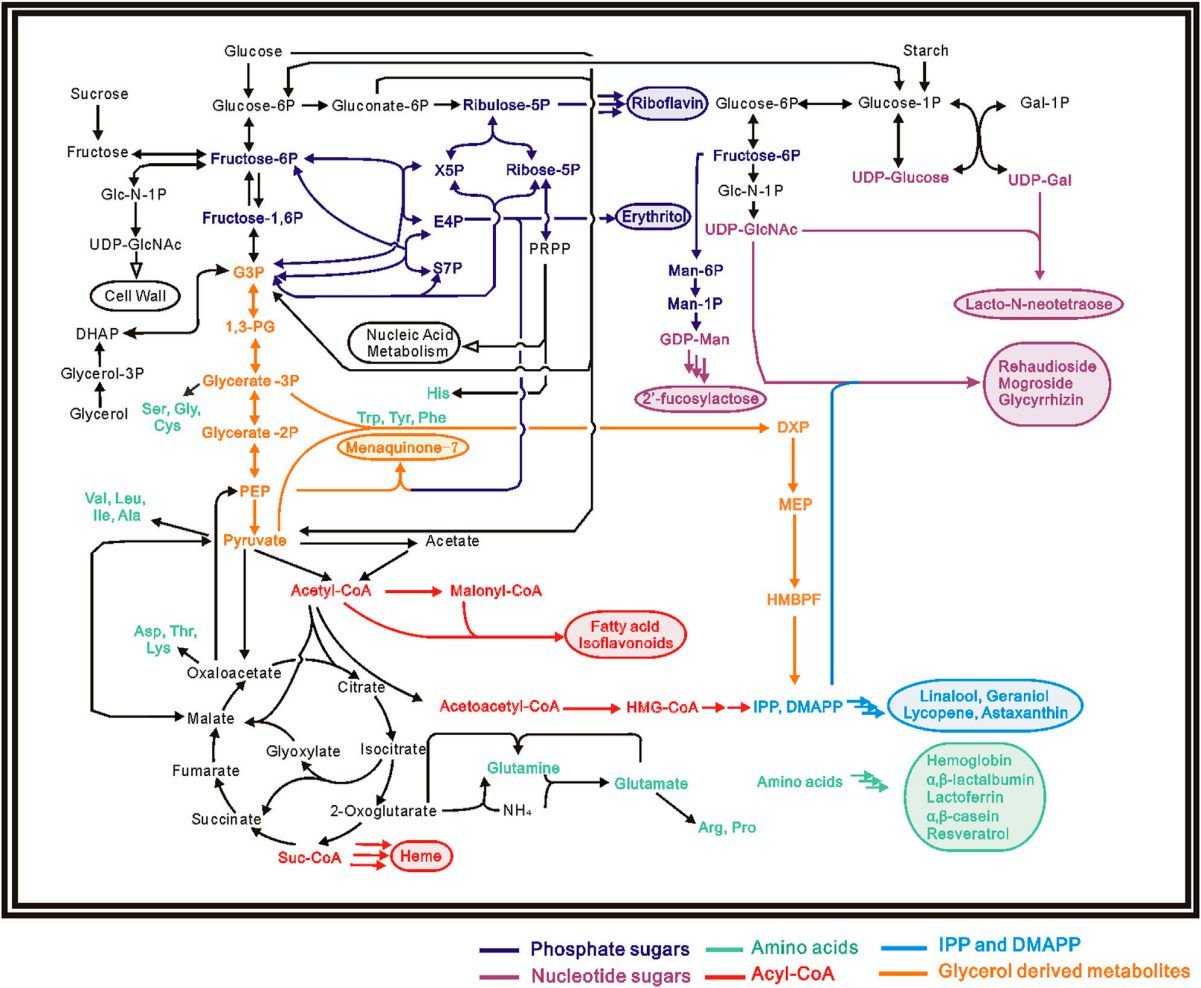
Pathway map for several biosynthetic food components, products are shown in the same color with their corresponding precursors.

Although the biosynthetic pathways are different, the basic building block of food components (proteins, carbohydrates, fatty acids and vitamins) all come from molecules in primary metabolism: such as embden–meyerhof pathway (EMP), tricarboxylic acid cycle (TCA), pentose phosphate (PP) pathway and amino acid biosynthesis (Hong et al., 2017). Energy and reducing power are also usually required for most of the biosynthetic process (Zhang et al., 2017). Figure 1; Table 1 showed current microbial produced food components and their biosynthetic precursors.

**TABLE 1:** Examples of biosynthetic food and their corresponding precursors.

Food type	Molecule synthesized by microbial cell factories	Precursors
Meat	Hemoglobin protein (α_2_β_2_)	Amino acids
	Heme	Acyl-CoA
	Fatty acids, Lipids	Acyl-CoA
Milk	α-lactalbumin	Amino acids
	β-lactoglubulin	Amino acids
	lactoferrin	Amino acids
	α-casein	Amino acids
	β-casein	Amino acids
Beer	Linalool	IPP and DMAPP
	Geraniol	IPP and DMAPP
Sweeteners	Rehaudioside	Nucleotide sugars, IPP and DMAPP
	Mogroside	Nucleotide sugars, IPP and DMAPP
	Glycyrrhizin	Nucleotide sugars, IPP and DMAPP
	Erythritol	Phosphate sugars
Nutritious supplements	Lycopene	IPP and DMAPP
	Astaxanthin	IPP and DMAPP
	Menaquinone-7 (vitamin B7)	Phosphate sugars, Glycerol derived metabolites
	2′-fucosylactose	Nucleotide sugars
	Lacto-N-neotetraose	Nucleotide sugars
	Riboflavin (vitamin B2)	Phosphate sugars
	Resveratrol	Amino acids, Acyl-CoA
	Isoflavonoids	Acyl-CoA

During the process of pathway engineering, precise quantitation of the precursors provides a clear indication to the next round of design-build-test-learn cycle, resulting in further engineering to achieve the final product. For example, during the process of engineering *Bacillus subtilis* for 2’–fucosylactose [Human milk oligosaccharides (HMOs)] production, GDP-L-fructose is a key precursor to the final product. The determination of GDP-L-fructose concentration was used to evaluate the possible effect of introducing the salvage pathway from *B. fragilis* and change of medium compositions on 2’–fucosylactose production, leading to next round of engineering for high yield 2’–fucosylactose production (Jieying et al., 2019). Another example is engineering *Escherichia coli* for carotenoids production by modular enzyme assembly methods, concentration of precursors such as acyl-CoA, isopentenyl diphosphate (IPP) and dimethylallyl diphosphate (IMAPP) was used as indicators to confirm that the metabolic network was changed by enzyme modulation towards carotenoids (Kang et al., 2019).

**FIGURE 2:**
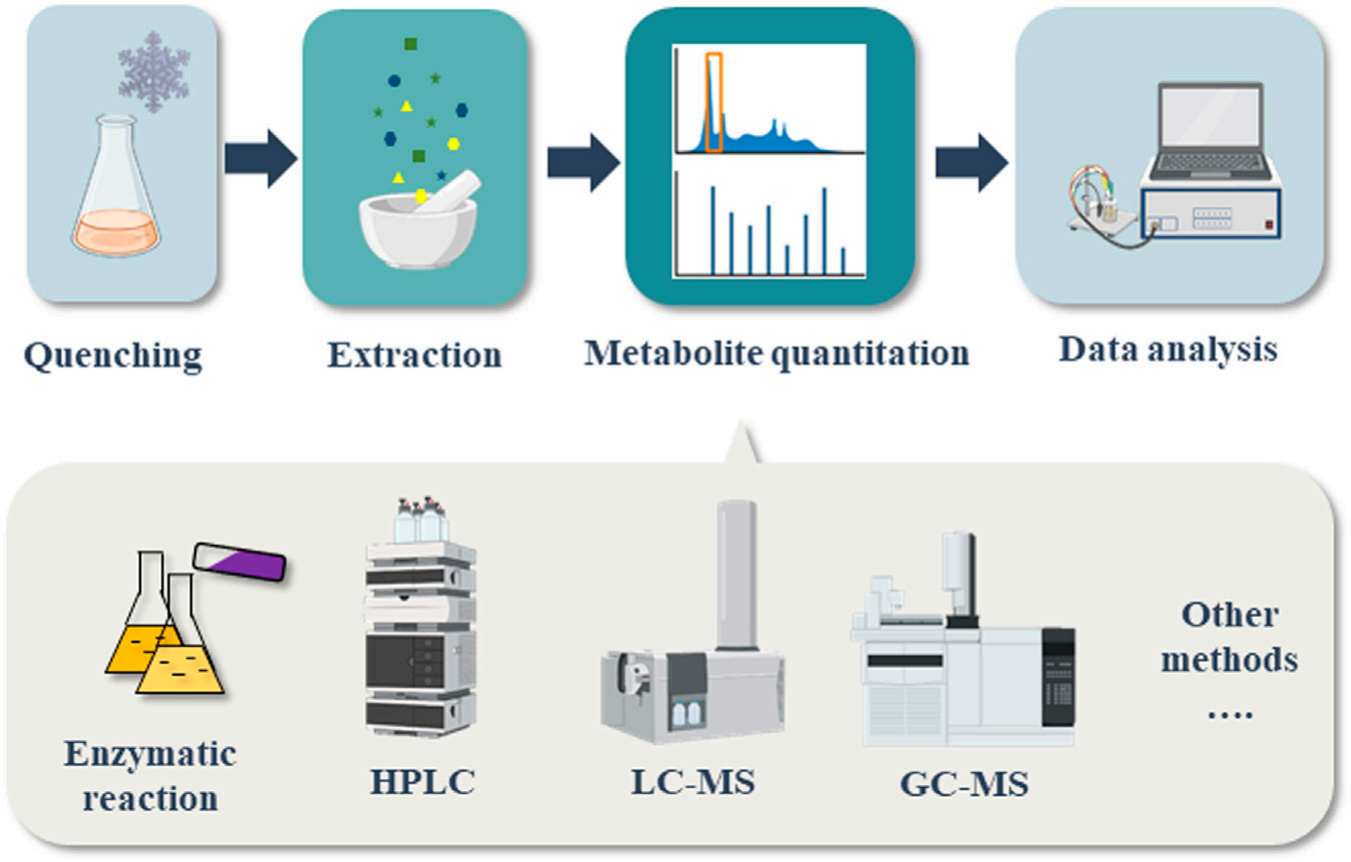
Flow chart of precursor determination process for biosynthetic foods.

Various quantification methods and instruments have been developed for quantitation of intracellular central metabolites. The procedure usually includes four steps: quenching of the samples for maintaining the metabolites concentration at specific time, extraction of intracellular metabolites, metabolites quantitation and data analysis (Figure 2) (Zhou et al., 2012). Several good reviews have mentioned about different quenching and metabolites extraction methods (van Gulik 2010; Vuckovic 2012; Causon and Hann 2016; Pinu et al., 2017). The specific analytical methods for determination of each kind of precursors have not been summarized individually. This review will emphasize on listing and discussing the quantitation methods for several important precursors in food biosynthesis. Among the various quantitation techniques have been developed, we will mainly discuss the analytical methods that can be easily carried out in every lab, namely the methods based on enzymatic reactions, HPLC, LC-MS and GC-MS. For a better understanding, the procedure of quenching and metabolites extraction is summarized briefly.

## A Brief Introduction on Quenching and Extraction Methods for Metabolites Quantitation

In cell, many metabolites are in a state of fast conversion into other chemicals catalyzed by various enzymes. In order to estimate the concentration of metabolites under specific condition precisely, the interference of enzymes must be removed as much as possible, which is called quenching. The enzymes must be inactivated in a short time, to avoid any changes of the metabolites. The most widely used method for quenching is cold methanol methods (Xu et al., 2016). For example, 60% methanol aqueous solution at −48°C is often used for fast quenching of the samples (Winder et al., 2008). The addition of cold methanol provides extreme cold environment and preventing cells from contacting oxygen and other nutrients, shutting down the metabolism and keeping the level of intracellular metabolites unchanged. Except for cold methanol method, other quenching methods such as fast vacuum filtration, direct heating (Hiller et al., 2007), glycerol–saline (Villas-Boas and Bruheim 2007) and flow cytometry (Wang et al., 2014) are also widely used in researches.

After quenching, intracellular metabolites need to be released from cells for quantitation. The purpose of chemical extraction is to disrupt cell membranes and harvest intracellular metabolites. Several metabolites extraction methods are used in researches, such as cold methanol extraction, boiling ethanol extraction, methanol and chloroform extraction, hot water extraction, potassium hydroxide extraction and perchloric acid extraction, each designed for extraction of specific kinds of metabolites (Park et al., 2012). The methods are usually in combination of solvent extraction and physical strike, such as sonication, liquid nitrogen grounding, freeze-thaw cycle and bead beating (Sasidharan et al., 2012). Cold methanol extraction seems to be more favored in many organisms, such as *E. coli* and *S. cerevisiae*, as most metabolites are extracted with high analytical stability (Maharjan and Ferenci 2003; Villas-Bôas et al., 2005; Winder et al., 2008). For the Gram-positive bacteria such as actinomycetes, liquid nitrogen grounding combined with cold methanol extraction seems to have a better effect because of the thick cell membrane (Zhao et al., 2013).

## Determination of Natural Products Precursors

In this section, we will discuss the quantitation methods for several important precursors that are involved in food biosynthesis. Namely, Acyl-CoAs, amino acids, nucleotide sugars, phosphate sugars, glycerol derived metabolites, IPP and DMAPP. The quantitation methods for NTPs and NAD(P)H levels are discussed in detail.

### Acyl-CoAs

Acyl coenzyme are main precursors for natural products such as fatty acids (Zhu et al., 2017) and heme (C4 pathway) (Xin et al., 2018), components in meat analog. In addition, they also serve as building blocks for biosynthesis of nutritious supplements such as resveratrol (Pannu and Bhatnagar 2019) and isoflavonoids (Liu et al., 2021). Among them, acetyl-CoA, malonyl-CoA and methymalonyl-CoA are most widely used substrates, while other acyl derived CoAs such as ethylmalonyl-CoA, methyoxymalonyl-CoA, chloroethylmalonyl-CoA, allylmalonyl-CoA and hexylmalonyl-CoA also took part in the biosynthesis of some compounds (Ray and Moore 2016).

The best enzymatic reaction for acetyl-CoA determination uses aromatic amines as substrate. Arylamine transacetylase catalyzes the reaction of *p*-nitroaniline (λ_max_ = 388 nm) and acetyl-CoA into nitroacetanilide (λ_max_ = 318 nm, absorption at 405 nm is close to zero) and CoASH. The amount of acetyl-CoA can be easily determined by following the reaction through absorbance measurement at 405 nm (Decker 1965). This method can determine less than 10 μmol/L of acetyl-CoA (Decker 1965). John. R. Williamson et al. described two other methods for acetyl-CoA determination (Williamson and Corkey 1969). One method uses reactions catalyzed by α-ketoglutarate oxidase and phosphotransacetylase. In the reactions, Acetyl-CoA and α-ketoglutarateis are converted into succinyl-CoA and CO_2_, generating one molecule of NADH. The amount of Acetyl-CoA will be determined by following the increase of absorbance of NADH. The other method uses citrate synthase and malate dehydrogenase. Citrate synthase catalyzes acetyl-CoA and oxaloacetate into citrate, and malate dehydrogenase converts malate into oxaloacetate coupling with the generation of 1 molecule NADH. The amount of acetyl-CoA can also be determined by followed the changes of NADH absorbance. However, the formation of NADH is not in stoichiometric with the consumption of acetyl-CoA, which needs a further correction for valid data (Williamson and Corkey 1969). In addition to the traditional enzymatic quantitation, commercialized kits for fast and precise determination of intracellular acetyl-CoA are also available nowadays.

Detection of acyl-CoA using high performance liquid chromatography (HPLC) are elucidated in researches (Boynton et al., 1994; Shibata et al., 2012; Tsuchiya et al., 2014; Shurubor et al., 2017). A C18 column and saline buffer as mobile phase is often used for clear separation of CoAs with other metabolites (Boynton et al., 1994; Shurubor et al., 2017). Shurubor *et. al.*, has developed a simple and sensitive method based on HPLC-UV to determine CoA and acetyl-CoA. A RP-C18 ((150 × 3 mm, 3 µm) analytical column and mobile phase consisted of monosodium phosphate, sodium acetate and acetonitrile was applied. The detection wavelength of the UV detector was set at 259 nm. Both CoA and acetyl-CoA elute within 10 min, and can be separated well. The limit of detection (LOD) for CoA and acetyl-CoA are 0.114 and 0.36 pmol per inject (Shurubor et al., 2017).

High performance liquid chromatography tandem mass spectrometry (LC-MS/MS) has become a widely used instrument for accurate qualitative and quantitative analysis. Determination of intracellular acyl-CoAs using LC-MS/MS has been described in many studies (Armando et al., 2012; Gilibili et al., 2011; Gotoh et al., 2015; Hasegawa et al., 2017; Kato et al., 2012; Kawaguchi et al., 2015; Seifar et al., 2013; Xia et al., 2013). The parameters of LC part are similar with HPLC detection, except for the mobile phase of saline buffer is replaced by volatile acetate amnion buffer, and flow rate is decreased below 0.5 mL/min, as a protection for mass spectrometry (Armando et al., 2012). For MS settings, multiple reaction monitoring (MRM), also known as selected reaction monitoring (SRM), is usually applied in LC-MS/MS based intracellular metabolites quantitation. In MRM mode, the mass of a precursor ion and a product ion for a specific metabolite was selected (Table 2), resulting in accurate quantitation without interference of other metabolites (Luo et al., 2015). Gilibili et al. adopted a method based on LC-MS/MS for quantitation of acetyl-CoA and malonyl-CoA. A monolithic RP-18 column was applied, 5 mm ammonium formate—acetonitrile (30:70, v/v) soulution was used as mobile phase. For MS settings, positive multiple reaction monitoring (MRM) mode was applied for detection of acetyl-CoA and malonyl-CoA. The total run time per sample is 3 min. The lower limit of quantification for acetyl-CoA and malonyl-CoA is 1.09 ng/mL (Gilibili et al., 2011). The presence of isomers (such as methymalonyl-CoA and succinyl-CoA) may bring difficulties for MS separation. This problem can be solved with the presence of HPLC. For example, with 400 mmol/L aqueous HCOONH_4_ and acetonitrile as mobile phase, Gotoh et al. successfully separated methymalonyl-CoA from succinyl-CoA using LC-MS/MS, peaks of the two compounds appeared on different retention time without interrupting each other. The lower limits of quantitation of methymalonyl-CoA and succinyl-CoA are 0.003 μmol/L and 0.01 μmol/L, respectively (Gotoh et al., 2015).

**TABLE 2:** Ion-pairing for MRM-based LC-MS/MS detection of some metabolites.

Compound	Parent ion	Product ion	Reference
Acetyl-CoA	810	303	Armando et al. (2012)
Malonyl-CoA	854	347	Armando et al. (2012)
Methylmalonyl-CoA	868	361	Armando et al. (2012)
Alanine	90	44	Le et al. (2014)
Arginine	175	70	Le et al. (2014)
Aspartate	134	88	Le et al. (2014)
Glutamate	148	102	Le et al. (2014)
Histidine	156	110	Le et al. (2014)
Leucine	132	86	Le et al. (2014)
Tryptophan	205	188	Le et al. (2014)
UDP-Glucose	565	323	Le et al. (2014)
UDP-GlcNAc	606	385	Le et al. (2014)
Glucose-6-phosphate	259	79	Luo et al. (2007)
Glyceraldehyde-3-phosphate	169	97	Luo et al. (2007)
Ribose-5-phosphate	229	97	Luo et al. (2007)
Sedoheptulose-7-phosphate	289	97	Luo et al. (2007)
Glucose-1-phosphate	259	241	Luo et al. (2007)
Erythrose-4-phosphate	199	97	Luo et al. (2007)
Phosphoenolpyruvate	167	79	Luo et al. (2007)
Pyruvate	87	43	Luo et al. (2007)
IPP	245	177	Henneman et al. (2011)
DMAPP	245	159	Henneman et al. (2011)
ATP	506	159	Luo et al. (2007)
GTP	522	424	Luo et al. (2007)
ADP	426	79	Luo et al. (2007)
NADH	664	79	Luo et al. (2007)
NADPH	744	408	Luo et al. (2007)

### Amino Acids

Amino acids are not only the building blocks of proteins (hemoglobin protein, *α*, β—casein, etc.), but also precursors of many natural products. For example, tyrosine is the precursor for resveratrol biosynthesis (Pannu and Bhatnagar 2019). Except for the 26 commonly seen amino acids, S-Adenosyl-L-Methionine (SAM), also acts as a methyl group donor for many natural products biosynthesis (Chen et al., 2016). As the precursor for protein synthesis, the concentration of most amino acids is comparably higher than other intracellular metabolites, therefore they are comparably easier to be quantified.

The amount of amino acids can be quantified with corresponding decarboxylation or transamination reactions (Bergmeyer et al., 1974). For L-alanine and L-aspartate, the amino group can be transferred to α-oxoglutarate by transaminases, generation L-glutamate and the corresponding carboxylic acids, which can further be reduced with the consumption of NADH. The level of amino acids can then be determined after measuring NADH consumption at 340 or 366 nm. L-arginine, L-tyrosine, L-ornithine, L-glutamate, L-histidine and L-lysine can be determined by corresponding decarboxylation reactions, CO_2_ was generated by removing the carboxyl group of the amino acids. The levels of amino acids can be quantified by measuring the generated CO_2_ content. For example, L-lysine can be catalyzed by L-lysine decarboxylase into cadaverine and CO_2_ at pH 6.0. The L-lysine content can be determined by quantifying CO_2_ emission with a manometer (Bergmeyer et al., 1974).

Since most amino acids have no UV absorption, it is impossible to detect amino acids using HPLC with UV spectrometry. The detection sensitivity will be largely improved by sample derivatization (Tian et al., 2014). Many reagents were available for derivatization of amino acids. Reagents such as o-phthaldialdehyde (OPA) (Ro and Hahn 2005), naphthalene-2,3-dicarboxaldehyde (NDA) (Tseng et al., 2009), fluorescein isothiocyanate (FITC) (Carlavilla et al., 2006), dansyl chloride (Giuffrida et al., 2009), and 9-fluoroenylmethyl chloroformate (FMOC) (Han and Chen 2007) and ninhydrin (de Paiva et al., 2013) are widely used for HPLC analysis (Lin et al., 2013), methyl chloroformate, heptafluorobutanol, trifluoroacetic anhydride and N,O-bis (trimethylsilyl) trifluoroacetamide (BSTFA) are usually used for GC-MS analysis (Kaspar et al., 2008; Waldhier M. C. et al., 2010; Kvitvang et al., 2011). New derivatization reagents are continually been reported (Otter 2012).

The most widely used HPLC techniques for amino acids quantitation is based on an ion-exchange chromatography followed by ninhydrin derivation and UV detection, which is also commonly called Amino Acid Analyzer (Le Boucher et al., 1997). Amino Acid Analyzer enables automatic separation, derivation and detection of amino acids with high accuracy and reproducibility. The lower limits of detection is less than 5 μmol/L (Le Boucher et al., 1997). Except for Amino Acid Analyzer, other HPLC methods are also used in chromatographic separation of amino acids (Callejón et al., 2008; Zhang et al., 2012; Shi et al., 2013). For example, Zhang et al. established a method based on HPLC to detect 23 amino acids in rat serum. The samples were treated with pre-column derivation using 2,4-dinitrofluorobenzene (DNFB), then went through HPLC analysis using a C18 (4.6 × 50 mm, 1.8 μm) column, the UV detection was set at 360 nm. Mobile phase was consisted of 10 mm ammonium acetate solution (A), acetonitrile (B) and methanol (C), a ternary gradient elution was carried out for a better resolution. All 23 amino acids eluted within 10 min and good separation was achieved. The lower limits of quantification is 5 μmol/L (Zhang et al., 2012). In addition to UV detector, photodiode array or fluorescence detector is also used in studies according to the types of derivation (Schwarz et al., 2005).

LC-MS/MS was widely used for fast, simple and reliable quantitation for amino acids (Petritis et al., 2000). Wang et al. determined leucine, isoleucine and valine by HPLC-MS/MS, using an EZ:faast^TM^ amino acid analysis-mass spectrometry column (250 × 3.0 mm, 4 μm). The mobile phase consist of 10 mm ammonium formate containing water (A) and methanol (B). Gradient flow was used for better resolution. MS was operated in positive ion electrospray mode and the amino acids were detected by MRM mode. The method was found to be sensitive and reproducible, and the lower limits of quantitation is 0.01 μg/mL (Wang et al., 2015). Quantitation of amino acids without derivation is simple and time saving. However, because the molecule weight of most amino acids are below 200, the signal acquired by MS spectrometry may be influenced by background noise or ion suppression. Stable isotope labelled amino acid as an internal standard (IS) can be used to solve this problem (Piraud et al., 2005a; Piraud et al., 2005b). For example, Piraud et al.(2005a) described a method using reversed-phase LC-MS/MS for the analysis of amino acids. The ion-pairing reagent tridecafluoroheptanoic acid was added into the mobile phase for a better separation, and a gradient of acetonitrile was used for the elution of the most compounds. Stable isotope labelled (SIL) AA was used as internal standard. For several amino acids, a good resolution was achieved, and the lower limits of quantitation were found to be about 0.1 μmol/L.

GC-MS is also widely used for amino acids determination (Stalikas and Pilidis 2000; Zampolli et al., 2007; Kaspar et al., 2008; Kaspar et al., 2009; Waldhier MC. et al., 2010; Kvitvang et al., 2011). Derivation to make stable and volatile compounds is necessary for GC analysis. Alkylation reagents, especially chloroformates, have been used frequently in studies (Kvitvang et al., 2014). For example, Kvitvang et al. (2014) used methyl chloroformate (MCF) for the derivation of samples for GC-MS analysis. The method covers over 60 metabolites. Metabolite with amino acid or carboxylic acid functional group yields a stable and volatile MCF derivative can be detected in this method. The low limits of quantitation is down to picomole range injected on column.

S-Adenosyl-L-Methionine (SAM), one of the nonprotein amino acid, is an important precursor in many natural product biosynthesis. Along with folic acid and vitamin B_12_, SAM serve as methyl donor in many cellular reactions (Zhou et al., 2002). It is necessary to determine SAM concentration in order to investigate whether the methyl group supply is sufficient in natural product biosynthesis (Ning et al., 2017). Similar with other amino acids, the quantitation methods for SAM is mainly carried out using HPLC (Zhou et al., 2002; Okamoto et al., 2003; Han et al., 2015; Hayakawa et al., 2016) and LC-MS/MS (Owens et al., 2015; Arning and Bottiglieri 2016; Manzanares-Miralles et al., 2016). The difference is SAM can be traced by UV detector directly at 254 nm, no derivation steps are needed (Han et al., 2015). For example, Han et al. determined the concentration of SAM in *Corynebacterium glutamicum* using HPLC. A Thermos BioBasic SCX column (4.6 × 250 mm) was used, mobile phase consisted of 100 mm ammonium formate, and UV detector was set at 254 nm. SAM was successfully detected and quantified. The concentration of SAM was calculated according to the peak area of different dilutions of SAM standards (Han et al., 2015). LC-MS/MS is also been used for SAM quantitation. Manzanares-Miralles et al. (2016) used LC-MS for the detection of SAM in *Aspergillus niger.* The samples were analyzed by LC–MS/MS using a porous graphitized carbon (PGC) chip on a 6,340 Ion-trap LC Mass Spectrometer*.* SAM levels were determined as Relative Quantification (RQ) by LC–MS analysis.

### Nucleotide Sugars

Nucleotide activated sugars take part in all kinds of glycosylation reactions in cell. They are not only the building blocks for the bacterial cell wall, but also the supply of glycosyl moiety for many metabolites (Ramm et al., 2004). The most widely used nucleotide sugars for natural products biosynthesis are NDP-Glucose and NDP-N-Acetylglucosamine (NDP-GlcNAc). For example, UDP-Glucose is responsible for biosynthesis of next-generation sweetners rehaudioside (Olsson et al., 2016), and NDP-GlcNAc is the precursor for human milk oligosaccharides (HMOs) Lacto-N-neotetraose (Dong et al., 2019).

The intracellular UDP-glucose can be determined by two enzymatic methods (Mills and Smith 1965). The first one uses Uridyl transferase, phosphoglucomutase and glucose-6-phosphate dehydrogenase. They catalyze the reaction from UDP-glucose, pyrophosphate and triphosphopyridine nucleotide (NADP) into 6-phosphogluconic acid, UTP and NADPH. The reaction can be followed by the absorption of NADPH at 340 nm. This reaction is specific for UDP-glucose (Mills and Smith 1965). The other method is based on the reaction catalyzed by UDPG dehydrogenase, UDP-glucose and 2 molecules of diphosphopyridine nucleotide (NAD) are transformed into UDP-glucuronic acid and 2 molecules of NADH. The reaction is irreversible. UDP-glucose will be determined according to the amount of generated NADH. This method can’t distinguish among UDP-glucose, UDP-galactose and UDP-GlcNAc, intracellular glucose-6-phosphate can also be an interference (Mills and Smith 1965; Lowry and Passonneau 1972).

HPLC methods have been developed for separation and quantitation of nucleotide sugars. Since most nucleotide sugars have UV absorption at around 260 nm, UV detector is usually applied for detection of these compounds. Two kinds of HPLC-based methods have been used for nucleotide sugars determination (Ramm et al., 2004): the high performance anion exchange chromatography (HPAEC) and ion-pair reverse phase high performance chromatography (RP-HPLC). For HPAEC, an anion exchange chromatography column is used, the mobile phase are NaOH/NaAc and NaAc water solutions (del Val et al., 2013; Tomiya et al., 2001). For example, Tomiya et al used HPAEC method for the quantification of nucleotide sugars from mammalian cells. The analysis was carried out using a CarboPac PA-1 column and mobile phase consisted of 1 mm sodium hydroxide (E1) and 1 m sodium acetate in 1 mm sodium hydroxide (E2). Sugar nucleotides were detected at 260 nm. A good resolution was achieved using HPAEC method. The lower limit of quantification was about 1 pmol/injection (Tomiya et al., 2001). For RP-HPLC, a C18 column is usually used (Ying et al., 2009), and mobile phase are usually phosphate aqueous solution and acetonitrile or methanol, with the addition of ion-pair reagents such as tetrabutylammonium hydrogensulphate (Nakajima et al., 2010; Ishibashi and Hirabayashi 2015), trietylamine, tripropylamine and tributylamine (Ramm et al., 2004). Ishibashi et al. employed RP-HPLC for the determination of nucleotide sugars in human cells. An ODS-3 column (4.6 × 150 mm, 3 μm) was used for separation of compounds. Ion-pairing reagent tetrabutylammonium hydrogen sulfate was added into potassium phosphate buffer, resulting buffer C. 70% of buffer C and 30% of acetonitrile was mixed to make buffer D. Gradient elution of buffer C and D was applied at a flow rate of 1.0 mL/min. UV detector was set to a wavelength of 254 nm. The amount of UDP-Gal, UDP-Glc, UDP-GalNAc and UDP-GlcNAc have been successfully quantified (Ishibashi and Hirabayashi 2015). In addition to UV detector, pulsed amperometric detector (PAD), photodiode array detector (PDA) have also been adopted for nucleotide sugars detection (Marcellin and Abeydeera 2009; Franke et al., 2015).

LC-MS/MS methods for determination of nucleotide sugars are mainly developed based on the HPLC methods of HPAEC and RP-HPLC (Rejzek et al., 2017). Negative modes with either full scan or MRM mode are selected for MS settings (Turnock and Ferguson 2007). For anion exchange chromatography, high salt mobile phase has to be replaced by PH gradient elution, and the NaOH solution be removed after column (Veltkamp et al., 2006; Alonso et al., 2010). For example, Alonso et al quantified cell wall precursors using this method. The metabolites were separated by an IonPac AS11 (25 × 2 mm) column at a flow rate of 0.35 mL/min. 0.5 mm NaOH (A) and 50 mM NaOH (B) were used as mobile phase. After column, the eluent went through an anion self-regenerating suppressor ASRS 300 (2 mm, Dionex) for the dilution of NaOH. MS was tuned at negative ion mode and MRM. 16 hexose-phosphate and nucleotide sugars were separately quantified (Alonso et al., 2010). For the ion-pairing RP-HPLC, the volatile ion-pairing reagents such as triethylammonium acetate or tripropylammonium acetate are be used v. For example, Turnock et al. determined sugar nucleotide pools of *Trypanosoma brucei*, *Trypanosoma cruzi* and *Leishmania major*. 0.5–4% acetonitrile in 20 mM triethylammonium acetate buffer (pH = 6) was used as mobile phase. A C-18 column was used. MS was operated in negative ion mode and analysis was carried on in MRM mode. The method successfully quantified the intracellular sugar nucleotides, and had a lower limits of detection at about 1 pmol/injection (Turnock and Ferguson 2007).

### Phosphate Sugars

Except for nucleotide sugars, another sugar source for glycosylation reactions are phosphate sugars. Phosphate sugars includes glucose-6-phosphate, glucose-1-phosphate, and the sugars involved in pentose phosphate pathway such as sedoheptulose-7-phosphate, erythrose-4-phosphate and ribose-5-phosphate. Phosphate sugars are the precursors for the biosynthesis of many natural products. For example, the sweetener erythritol is biosynthesized from erythrose-4-phosphate (Rzechonek et al., 2018), while riboflavin (vitamin B2) comes from ribulose-5-P (Nielsen and Bacher 2009), etc.

Different phosphate sugars can be determined after transforming into glucose-6-phosphate or glyceraldehyde-3-phosphate by corresponding transketolase or transaldolase (Bergmeyer et al., 1974). Glucose-6-phosphate can be determined by the reaction catalyzed by glucose-6-phosphate dehydrogenase, in which glucose-6-phosphate and NADP are converted into 6-phosphogluconic acid and NADPH. The reaction is followed by measuring OD at 340 nm, as the reduction of NADPH. Glyceraldehyde-3-phosphate reacts with NAD in the presence of arsenate, generating glycerate-3-phosphate and NADH. The reaction can also be followed by changes of OD at 340 nm, as the generation of NADH (Bergmeyer et al., 1974).

Determination of phosphate sugars by HPLC is challenging, since most phosphate sugars are highly hydrophilic and having no UV absorption. Alternatively, detectors such as pulsed amperometric detector (PAD) (Marcellin and Abeydeera 2009), amperometric detector (Qian et al., 2008) or aerosol detector (Hinterwirth et al., 2010) are adopted in researches. Hinterwirth et al. (2010) has established a method for the separation of sugar phosphates using HPLC. The HPLC method consisted a mixed mode chromatography with reversed-phase/weak anion-exchangers and charged aerosol detector. The method enabled almost complete resolution for a mixture of six hexose phosphates.

For a more precise detection and quantification capacity, mass spectrometry coupled with HPLC are used for determination of phosphate sugars (Feurle et al., 1998; Sekiguchi et al., 2005; Antonio et al., 2008; Kato et al., 2012; Xia et al., 2013; Qiu et al., 2016). Hydrophilic columns and ion-pairing reagents adding mobile phase are often applied for a better resolution. Antonio et al. used LC-MS/MS to detect phosphate sugars in *Arabidopsis thaliana* leaf tissue. A ZIC-HILIC column (150 × 2.1 mm, 3.5 μm) was used for separation. Mobile phase composed of 0.1% (v/v) formic acid (FA) in acetonitrile (A) and 0.1% FA in 5 mM ammonium acetate. MS was operated in the negative ion mode. A full scan mode over the scan range m/z 50 to 1,000 was applied. The method enabled separation and detection of eight sugar related compounds in less than 15 min. Limits of detection is 2.0 μmol/L for sugar phosphates (Antonio et al., 2008). Luo et al. described a method for simultaneous determination of multiple intracellular metabolites, including phosphate sugars by LC-MS/MS. A good resolution was achieved by use of the volatile ion pair modifier tributylammonium acetate (TBAA) in the mobile phase. 29 metabolites including sugar phosphates, nucleotides and carboxylic acids were separated on a C18 revered-phase column. The limits of detection for metabolites were mostly below 60 μmol/L (Luo et al., 2007). Qiu et al. compared different column, mobile phase and MS scan mode on simultaneous determination of 25 metabolites, including phosphate sugars. The NH2P-50 2D (2.0 × 150 mm, 5 μm) column, and the mobile phase of 1.5 mmoL/L ammonium bicarbonate and 0.1% concentrated ammonia in aqueous solution was shown to give best resolution for separation of all metabolites. The author also showed Full scan MS mode was better than the MRM mode for simultaneous detection of multiple metabolites. The calibration curved of this method showed good linearity within the range of 1—10,000 μg/L (Qiu et al., 2016).

GC-MS is also used for determination of phosphate sugars (Cipollina et al., 2009; Spegel et al., 2013; Liu et al., 2015; Uifalean et al., 2016; Xu et al., 2016; Shen et al., 2017). Sample derivation are usually required, the reagents such as methoxyamine and N-methyl-N-trimethylsilyl trifluoroacetamide are usually applied for better volatility of the chemicals (Spegel et al., 2013; Liu et al., 2015; Uifalean et al., 2016; Xu et al., 2016). For example, Spegel et al. determined intracellular metabolites in β-cells by GC-MS. The samples were treated for derivation with methoxyamine hydrochloride and N-methyl-N-trimethylsilyl trifluoroacetamide after extraction. The separation was performed on a 30 × 0.25 mm DB5-MS column with a phase thickness of 0.25 μm. The ionization energy was set to 70 eV and the data acquisition rate was 20 Hz with a scanning range of 50–800 m/z. The data were treated using MATLAB and HMCR. Peak identification was performed using NIST MS search 2.0. The study successfully determined metabolites concentration in the control of insulin release in cell. The changes in ribose 5-phosphate and other metabolites were well recorded (Spegel et al., 2013).

### Glycerol Derived Metabolites

Glycerol derived metabolites not only take part in the biosynthesis of varies natural products [such as Menaquinone-7 (vitamin B7)] (Cui et al., 2019), but also as a precursor for the biosynthesis of other precursors (such as acyl-CoAs, IPP, and DMAPP, etc.). In these compounds, the direct precursors of the glycerol moieties are 1,3-biphosphoglycerate (1,3-BPG), 2 (or 3)- phosphoglycerate [2 (or 3)-PG] or phosphoenolpyruvate (PEP).

It is difficult to determine the intracellular pool of 1,3-BPG, as 1,3-biphosphoglycerate spontaneously decomposes to give 3-phosphoglycerate and inorganic phosphate. The amount of 1,3-biphosphoglycerate is very low and its estimation is hardly possible. The only way to determine the concentration of 1,3-biphosphoglycerate is by enzymatic reaction (Bergmeyer et al., 1974; Inoue et al., 1987), however the accuracy is not guaranteed.

While few studies have reported determination of glycerol phosphates with HPLC, the concentration of 2-PG, 3-PG and PEP can still be determined by MS-aided techniques. These compounds are often quantified along with other metabolites such as phosphate sugars and carbolic acids in metabolome related researches (Kato et al., 2012; Spegel et al., 2013; Rehberg et al., 2014; Nishino et al., 2015; Qiu et al., 2016; Uifalean et al., 2016; Boone et al., 2017). The signals of 2-PG and 3-PG are not easily separated by liquid chromatography, and better resolution is achieved by optimization of the column and mobile phase. Qiu et al. reported that with the use of a NH2P-50 2D column, and mobile phase consisted of ammonium bicarbonate and 0.1% ammonia added acetonitrile aqueous solution or methanol aqueous solution, a complete separation of 2-PG and 3-PG can be achieved (Qiu et al., 2016).

### IPP and DMAPP

Isopentenyl diphosphate (IPP) and dimethylallyl diphosphate (DMAPP) are the common precursors of terpenes, a large natural products family for biosynthesizing of nutritious supplements carotenoids, including the famous molecule such as lycopene (Ma et al., 2019), β-carotene (Kim et al., 2008), astaxanthin (Park et al., 2018), etc. The biosynthesis of terpenes starts with the condensation of IPP and DMAPP, which are isomers to each other.

Few studies are present for the detection of IPP and DMAPP. Before the year of 2000, most quantitation studies were done using radio labelled precursors and radio detector for the quantitation of IPP and DMAPP (Bruenger and Rilling 1988; McCaskill and Croteau 1993; Lange et al., 2001). Then methods based on liquid chromatography coupled tandem mass spectrometry was developed, and determination of terpene precursors become easier (Henneman et al., 2008; Jauhiainen et al., 2009; Henneman et al., 2011). Henneman et al. used HPLC-MS/MS to quantify IPP/DMAPP pool along with six other intermediates in terpene biosynthesis. The samples were separated on a C18 column (4.6 mm × 50 mm, 3 μm) eluted using solution A (20 mm NH_4_HCO_3_, 0.1% triethylamine) and solution B (acetonitrile aqueous solution, 0.1%triethylamine). The analyze time is 12 min per sample. Mass spectrometry was tuned on negative MRM mode. Most metabolites had been successfully quantified, and the lower limit of quantitation of IPP/DMAPP is 0.42 μmol/L (Henneman et al., 2008). The only problem of the method above is its inability to separate the isomer IPP from DMAPP. Jauhiainen et al. solved this problem by detecting different MS2 spectra of IPP and DMAPP, based on different signal intensity ratio of the MS2 spectra of the isomers. In detail, the fragment ion m/z 177, formed by pyrophosphate group cleavage, was used for detection of IPP. While the fragment ion m/z 159 was more suitable for detection of DMAPP. After data calibration with IPP and DMAPP standards, both the concentration of IPP and DMAPP were quantified successfully (Jauhiainen et al., 2009). Tong et al. developed a method based on enzymatic reaction followed HPLC with fluorescence detection to determine the basal level of IPP in untreated cells (Tong et al., 2013). In the method, IPP and farnesyl diphosphate (FPP) can be catalyzed by geranylgeranyl diphosphate synthase into geranylgeranyl diphosphate (GGPP), which was further conjugated on a fluorescently labelled peptide. The resulted peptide can be determined using HPLC with a fluorescence detector. This method is specific for detection of IPP without the interference of DMAPP. The lower limit of detection reached about 5 pg (0.017 pmol) per injection.

### Others

Except for the direct precursors described above, the biosynthesis of natural products always require energy and reducing power to complete a series of reactions. The successful proceeding of reactions depends on the intracellular level of the energy and reducing power donors. Those chemicals includes nucleotide phosphates (ATP, GTP, TTP, dTDP, etc.) and adenine dinucleotides (NAD(P)H and FADH). Herein, we will take ATP and NAD(P)H as examples to discuss the quantitation methods of energy and reducing power donors.

### ATP

The most common principle for ATP determination by enzymatic reactions is the luciferase reaction (Strehler 1974; Yang et al., 2002; Gorman et al., 2003; Gorman et al., 2007). Luciferase catalyzes luciferin and ATP into adenyl-luciferin, whereas adenyl-luciferin can be oxidized into adenyl-oxyluciferin by the oxygen in atmosphere, in the same time light emission happens (Strehler 1974). The emission of light can be followed by a spectrometer. The quantitation of ADP and AMP was done after conversion into ATP by pyruvate kinase or myokinase (Gorman et al., 2003). Because of the high sensitivity and specificity of luciferase, the reaction is widely used for ATP determination. Currently, commercialized kits have also been developed for determination of intracellular ATP based on different enzymatic reactions.

HPLC is widely used for determination of intracellular ATP (Kawamoto et al., 1998; Huang et al., 2003; Caruso et al., 2004; Zur Nedden et al., 2009; Zhou et al., 2012). Ionized reagents such as potassium phosphate are used as mobile phase. Huang et al. detected intracellular nucleoside triphosphate levels in cells by a reverse phase ion-pair HPLC. Separations were performed using a C-18 (150 × 4.6 mm, 3.5 μm) column. The mobile phase consisted of 10 mM tetrabutylammonium hydroxide, 10 mM KH_2_PO and 0.25% MeOH (A) and 5.6 mm tetrabutylammonium hydroxide, 50 mM KH_2_PO and 30% MeOH (B). The UV detector was set at 254 nm. High resolution of nine nucleoside triphosphate in 16 normal or tumor cell lines was achieved. The detection limits of ATP and ADP are 2.44 pmol and 1.39 pmol per injection, respectively (Huang et al., 2003). Fluorescence detector is also adapted sometimes. For example, Kawamoto et al. used HPLC with fluorescence detector to quantify ATP and related metabolites from rat caudal artery. The samples were derived with chloroacetaldehyde to produce high fluorescent signals. After separation, the samples were detected by RF-10A fluorescence detector. The wavelength for excitation and emission were set at 270/410 nm for ethenopurine derivatives and 285/395 nm for underivatized purines. The ethenopurine derivatives of ATP were separated within 15 min, and the lower limits of detection was 0.04 pmol per injection (Kawamoto et al., 1998).

LC-MS/MS is also widely used for ATP quantitation, the separation of the adenine nucleotides are usually accomplished by the addition of volatile ion-pairing reagents into the mobile phase (Qian et al., 2004; Cohen et al., 2009; Seifar et al., 2013; Zhang et al., 2014). MRM mode is applied for MS data collection. Zhang et al. established a method using LC-MS/MS for quantitation of endogenous adenine nucleotides in human plasma. The mobile phase was made based on ion-pairing reagents diethylamine (DEA) and hexafluoro-2-isopropanol (HFIP). The samples were separated by an aminopropyl (NH_2_) column. MS was tuned at negative-ion MRM mode. The method was reported to have satisfactory linearity, sensitivity, accuracy, reproducibility and matrix effects. The lower limits of quantitation is 2.0 ng/mL (Zhang et al., 2014).

### NAD(P)H

The featured fluorescence and absorption characteristic of NAD(P)H can be applied for its intracellular quantitation. The fluorescent excitation wavelength of NAD(P)H is 340 nm, and emission wavelength is 460 nm. The strongest absorption wavelength is at 340 nm. Based on these principles, spectroscopy or fluorescence spectroscopy can be applied for determination of intracellular NAD(P)H (Villette et al., 2006; Saliola et al., 2012). Saliola et al. examined intracellular NAD(P)H level with a FluoroMax-3 (Horiba Jobin-Yvon) spectrofluorometer. The excitation wavelength was set at 366 nm, and the emission spectra were recorded from 370 nm to 440 nm. The corresponding NAD(P)H concentration was calculated at the basis of a NADPH standard curve obtained from solutions of different concentrations (Saliola et al., 2012). NAD(P) can be converted into NAD(P)H by alcohol dehydrogenase (NAD) or by glucose phosphate dehydrogenase (NADP) [107], thus total and individual amount of NAD(P)H and NAD(P) can be calculated respectively (Wise and Shear 2006; Ogasawara et al., 2009). The spectroscopy based method is fast and easy. However, NADH and NADPH can not be distinguished directly, and other chemicals in cell may interrupted the detection result.

HPLC for NAD(P)H detection has promised an at least five times more sensitive result than enzymatic reaction (Ogasawara et al., 2009). Peaks of NADH and NADPH can be easily seperated by HPLC. Ion-pairing reverse phase HPLC with fluorescent detection (Ogasawara et al., 2009) or UV detection (Yoshino and Imai 2013) are usually applied. Ogasawara et al. detected NADPH and total NADPH (NADP + NADPH) in human red blood cells with HPLC equipped with an fluorescent detector (Ogasawara et al., 2009), the mobile phase consists of 5% methanol and 95% 0.1 M phosphate buffer, a reverse phase RP-C-18 (4.0 mm × 250 mm, 5 μm) column was applied for compounds separation. The excitation and emission wavelength was set at 340 nm and 460 nm, respectively. The peak of NADPH was detected, total amount of NADPH and NADP was monitored after converting NADP into NADPH by glucose phosphate dehydrogenase. The concentration of NADP(H) was calculated based on an NADPH standard curve (Ogasawara et al., 2009).

LC-MS based detection of NAD(P)H has also been widely used, giving a clear distinguish among reduced and oxidized formed NADH and NADPH and a much higher detection sensitivity (Ortmayr et al., 2014; Lu et al., 2017). Ortmayr et al. evaluated several analytical workflow for analyzing oxidized and reduced form of NADPH in the yeast *Pichia pastoris*. An optimal chromatographic separation is achieved with a silica-based C-18 (2.1 mm × 150 mm, 3 μm) column. The mobile phase consisted of 5 mM ammonium acetate (pH 6.0, A) and methanol (B). An excellent chromatographic resolution of NADP and NADPH was 4.9 min within a total run time of 12 min. LC-MS/MS in negative multiple reaction monitoring mode was used for detection and quantitation. The method was proved to be appropriate for quantitation of oxidized and reduced form of NADPH in yeast cells (Ortmayr et al., 2014).

## Discussion

In this review, methods commonly used for precursors quantitation were discussed in detail. Table 3 summarized the characteristics of the methods. Each method has advantages and limitations, and is suitable for different experimental purposes.

**TABLE 3:** Comparison of the methods mentioned in this article.

Methods	LOQ^a^ (μmol/L)	Throughput	Precursors can be quantified by this method	References
Enzymatic reactions	∼10	High	acyl-CoA, amino acids, nucleotide sugars, phosphate sugars, ATP, NAD(P)H	Decker (1965)
HPLC	5–100	Low	acyl-CoA, amino acids, nucleotide sugars, ATP, NAD(P)H	(Tomiya et al., 2001; Huang et al., 2003; Zhang et al., 2012; Shurubor et al., 2017)
GC-MS	5–100	Low	amino acids, phosphate sugars	Kvitvang et al. (2014)
LC-MS/MS	< 5	Low	acyl-CoA, amino acids, nucleotide sugars, phosphate sugars, glycerol derived metabolites, IPP and DMAPP, ATP, NAD(P)H	(Piraud et al., 2005a; Luo et al., 2007; Turnock and Ferguson 2007; Antonio et al., 2008; Henneman et al., 2008; Gilibili et al., 2011; Zhang et al., 2014; Gotoh et al., 2015; Wang et al., 2015; Qiu et al., 2016)

a LOQ, is the lower limits of quantitation, calculated from the corresponding research papers, the unit was normalized to μmol/L.

Enzymatic reaction provides fast and high-throughput detection, can be applied for detection of various metabolites, while the specificity is lower than other methods. The commercialized kits developed with high detection accuracy are very useful for high-throughput detection of specific compounds. Acyl-CoAs, ATP and NAD(P)H can be quantified easily using this method. HPLC provides a more specific detection, but having trouble for determination of metabolites with low UV absorption. The LOQ range of HPLC with UV detector is 10–100 μmol/L, which would be much improved when using fluorescence detector (to 4–5 μmol/L) (Tong et al., 2013) (Kawamoto et al., 1998). HPLC is a good choice for quantitation of nucleotide sugars. GC-MS analysis is also a choice of metabolites detection with higher specificity, but the metabolites must be volatile or can be derived into volatile molecule before analysis. GC-MS can be used for detection of amino acids and phosphate sugars. LC-MS/MS brings up to now most sensitive (LOQ < 5 μmol/L) and specific detections for metabolites, and can be applied to all the precursors mentioned in this article. However, the price for LC-MS/MS operation and maintenance are much higher than the former two. All the precursors mentioned in this review can be quantified by LC-MS/MS. Because of its high sensitivity and broad detection range, LC-MS/MS, or LC-MS/MS combined with GC-MS are widely used for metabolome analysis.

Apart from the methods above, many other methods have also been developed for metabolite quantitation. For example, there are many researches about metabolite determination using capillary electrophoresis (CE) (Cavazza et al., 2000; Boniglia et al., 2010; Tian et al., 2014; Crespo et al., 2015; de Souza Crespo et al., 2015). CE has emerged as a promising complementary technique to HPLC, as it has several advantages such as fast analysis, small sample volume demanding and low consumption of solvents (Tian et al., 2014). Capillary electrophoresis (CE) tandem mass spectrometry are also used in studies (Lehmann et al., 2000; Feng et al., 2008; Watanabe et al., 2015). Sometimes, NMR is also applied for detection of precursors (Sonnewald et al., 1994; Ramm et al., 2004). Those methods provides alternative choices for determination of natural products precursors according to different purposes.

In spite of great progress achieved in method development, there is still room for improvements. The biggest challenge is how to ensure both throughput and specificity. For enzymatic methods, evolution or engineering of enzymes with improved substrate binding affinity would increase the detection specificity, application of fluoresce instead of absorbance would also increase the sensitivity. MS detection provides most specific analysis up to now in spite of low throughput, to solve the problem, Matrix-Assisted Laser Desorption/Ionization-Time of Flight (MALDI-TOF) mass spectrometry (MS) has been developed which enabled high throughput detection of molecules, but mostly appropriate for detection of molecules with high molecule weight (biopolymers such as DNA, proteins, peptides and carbohydrates). If this technique can be improved for accurate detection of compounds with molecule weight of 50–1,000 Da, it will greatly improve the throughput of metabolites quantitation. There is also difficulty in detection and quantitation of some metabolites without commercial standards, therefore, free and comprehensive on line mass spectrometry databases for metabolomics analysis would promote the development of this area.
